# Supplementary material for: Patterns of Intron Gain and Loss in Fungi
Source: PLoS Biol. 2004 Nov 30;2(12):e422. doi: 10.1371/journal.pbio.0020422 (PMC532390; doi:10.1371/journal.pbio.0020422)
Supplement: Table S1 — Also available at http://genes.mit.edu/NielsenEtAl/. (4.3 MB ZIP). [file pbio.0020422.st001.zip › NielsenEtAl/html/1362.html]

AN0665.1.NCU01002.1.MG07035.1.FG10180.1


```
 CLUSTAL W (1.82) Multiple Sequence Alignments - Introns Inserted


Sequence 1: MG07035.1	297 aa
Sequence 2: FG10180.1	294 aa
Sequence 3: NCU01002.1	296 aa
Sequence 4: AN0665.1	302 aa
Alignment Length: 320 aa
Number Identitical Residues: 132 aa
Alignment Score (without introns) 6598


MG07035.1 	-MDPYSAEGE~LINIHNHFHQGQFQEVVDYDTSSLSPENALPARVLQLRSRIALGQAADV
NCU01002.1	MSDPFGAEAE~LINIESHFYQGQYQEVIDFDTTSFSADNALPARVLQLRARIALGQAEDV
FG10180.1 	-MDPYSTEGE~LINIHNYFHQGQYQEVVDFDTSSFSPDNALPVRVLVLRARLALGQAEDV
AN0665.1  	-MDPFSAEGE1LINIHNAFHQGQYQSVIDFNTSSFSPENALPARILKLRAQVALGNTDDV
          	  **:.:*.* ****.. *:***:*.*:*::*:*:*.:****.*:* **:::***:: **

MG07035.1 	LAEVKGESD--PELVVVGALAQQALGKADAAVAVVEKLAEASGDNP-VVQVVGGTVLQAA
NCU01002.1	LAEVAGEKK--PELEAVGALAQLKAGDAESAVETIEQLAATDAGEIGIVQVVGGTVLAAA
FG10180.1 	LDEVKGESE--PEFQALGALAELNLGKTDSAVQTIEKLVSSAADNT-TVQVIGGTVLQAA
AN0665.1  	LADIEGETEESPSLSAVKALAQFAAGNAEAATQLAQDLAENYPDDA-TVQVLGGTVLQNV
          	* :: **...:*.: .: ***:   *.:::*.   :.*.    .:   ***:*****  .

MG07035.1 	GKSEEALALLSKQQGNL1DA~VALTVQIHLALNRNDLALKEVKAARSWAQDSLLVNLAES
NCU01002.1	GKADEALALLQRHQGNL~EA~VALIVHIYLAQNRTELAVKEVASARRLAQDSLLFNLAES
FG10180.1 	GKSEEALALLTQHQGSL1DA~VSLIVQIHLQQNRTDLALKEVSAARRWAQDSLLVNLAES
AN0665.1  	GKSEEALALLGKHQGNL~EA2S-------------DLALKEVQAAKRWAQDSLLVNLAES
          	**::****** ::**.* :*               :**:*** :*:  ******.*****

MG07035.1 	WVGLRK0GGEAYQQAFYVFEELAQSASTASVRTLVAQAVAELHLGRTEEAQAALEQATQK
NCU01002.1	WVGLRL0GGDKYQQAFYVFEELAQAPATSSVRSLVSQAVAEIHLGRTEEAQAALDQALKK
FG10180.1 	WVGLRV0GGEKYQQAFYVYEELAQAPSTSSVRSLVSQAVCELHLGRTEEAQAALDQALEK
AN0665.1  	WVGMRV0GGEKYQSAFYVYEELASVDSTSAPLSIIGQAVAEIHLGRLPEAEAAISAALER
          	***:*  **: **.****:****.  :*::  :::.***.*:****  **:**:. * ::

MG07035.1 	DPNFAETVANLLVLKVITGASAD--------------EQT-----S2LLKKTAPDHPLLV
NCU01002.1	EPGFADAIANQLVWSVITGQDSA--------------EYK-----A2ALEQADPNHPFLT
FG10180.1 	DANNADAIANLLVLNVISGNKSD--------------EFT-----Q2KLQSAKPDHQYLA
AN0665.1  	YPNEAGLIANSIVLNVLIGKPTEDLEKYASLELSGRMEFTNGLRYR~RLQLVEPSHALLA
          	 .. *  :** :* .*: *  : . .. :: . :.  * ...      *: . *.*  *.

MG07035.1 	DLEEKSALFDKAATKYSAKVSTVPS
NCU01002.1	DLAEKSDLFDKAATKYKAKVSA---
FG10180.1 	DLEEKSALFDRAATKYSPKVSA---
AN0665.1  	DIQEKSDFFDTAAAKYSAKVSS---
          	*: *** :** **:**..***:
```
